# Mental Health Issues in the COVID-19 Pandemic and Responses in Bangladesh: View Point of Media Reporting

**DOI:** 10.3389/fpubh.2021.704726

**Published:** 2021-09-03

**Authors:** Mir Nabila Ashraf, Hannah Jennings, Nantu Chakma, Noshin Farzana, Md. Saimul Islam, Toufiq Maruf, M. M. Jalal Uddin, Helal Uddin Ahmed, David McDaid, Aliya Naheed

**Affiliations:** ^1^Initiative for Non-Communicable Diseases, Health System and Population Studies Division, International Centre for Diarrhoeal Disease Research, Bangladesh, Dhaka, Bangladesh; ^2^Department of Health Sciences, University of York and Hull York Medical School, Heslington, United Kingdom; ^3^Bangladesh Health Reporters' Forum, Dhaka, Bangladesh; ^4^Daily Kaler Kantha, Dhaka, Bangladesh; ^5^National Institute of Neurosciences & Hospital, Dhaka, Bangladesh; ^6^National Institute of Mental Health, Dhaka, Bangladesh; ^7^Department of Health Policy, Care Policy and Evaluation Centre, London School of Economics and Political Science, London, United Kingdom

**Keywords:** mental health stress, suicide, domestic violence, newspapers, COVID-19, media review

## Abstract

**Background:** The negative impact of COVID-19 on mental health has been reported by media throughout the world, although this role is not well-understood in low-and middle-income countries (LMIC). We examined the reporting of mental health issues during the COVID-19 pandemic in Bangladesh and initiatives undertaken to support mental health reported from the viewpoint of media.

**Methods:** We reviewed articles published in 10 local newspapers, including seven Bangla and three English newspapers, during the first year of the COVID-19 pandemic. News topics were identified through discussions among the team members, with searches across online newspapers and portals. Data extrapolated from newspapers were documented in an Excel spreadsheet. A mixed-method approach was used following a framework analysis for analyzing data. Recurring issues and commonly emerging topics were generated from the data. Descriptive statistics were applied for analyzing quantitative data.

**Results:** Between March 2020 and March 2021, we have identified 201 reports on mental health issues including 45 reports (22.4%) focused on stress due to the associated financial crisis, unemployment and loneliness, 50 reports (24.9%) of 80 apparent suicides linked to family issues, disharmony in conjugal relationships, harassment, sexual violence, emotional breakdown, financial crisis, and stigma due to COVID-19.There were 77 reports (38.3%) concerning domestic violence during the pandemic. Twenty-nine reports (14.4%) referenced actions taken by different organizations to address mental health issues in response to the pandemic in Bangladesh.

**Conclusion:** News coverage has the scope to highlight important issues that can emerge as a consequence of the COVID-pandemic, such as mental health, in a low resource setting. Capacity building of the media on the way to report mental health issues during emergency situations could be a useful strategy for more credible reporting on mental health issues during the COVID-19 pandemic for raising awareness of the public and policymakers about the negative consequences on mental health of the COVID-19 pandemic in Bangladesh. Adopting policies to support essential mental health care and promoting the local organizations to take timely public health measures will be imperative for averting the negative consequences of mental health due to the COVID-19 pandemic in Bangladesh.

## Introduction

Mental illness is a major contributor to the overall global burden of disease accounting for ~32.4% of years lived with disability and 13% of disability-adjusted life-years (DALYS) (1). In 2019 3.8% of the global population experienced depression and 4.1% anxiety disorders according to the Global Burden of Disease study (2). The issue is of growing significance in emerging economies such as Bangladesh where estimates of the prevalence of Common Mental Health Disorders, such as depression, are ~7% for adults over 20 years, 1% for children 10–14 years and 3% for the adolescents 15–19 years of age (2). Promoting good population mental health is critical to the development and sustainment of human and economic capital in any country (3). In contrast, poor mental health is associated with adverse social and economic outcomes, as well as poor physical health across the life course (4). The direct impacts of the current COVID-19 pandemic, as well as the policy response of governments, on mental health have been highlighted around the world, with the existing financial and social inequalities that impact on mental health reported to be exacerbated by the current COVID-19 pandemic (5).

Although there have been reports of some positive effects of the COVID-19 pandemic on family life and relationships, such as increased opportunities for families to spend time together (6), the pandemic has had many harmful impacts on psychological health. The pandemic changed a myriad of aspects of life and caused significant distress in high, middle and low-income countries across the world (7). For instance, the suddenness of the lockdown was associated with psychological distress, and it has been identified as a cause of panic disorders, anxiety, and depression in China (8, 9).

The first cases of COVID-19 in Bangladesh were identified on 8 March 2020 (10). With cases rising rapidly, the first nationwide lockdown was imposed on the 26 March 2020 (11). The pandemic changed a myriad of aspects of people's lives and caused significant distress in high, middle and low-income countries across the world (7).

Even with the roll out of vaccination programs, the primary public health measures for preventing transmission of the SARS-Cov-2 virus responsible for the COVID-19 pandemic involve isolation; the very thing that may lead to loneliness, anxiety, depression, anger, or fear and could trigger new or existing mental health issues (12, 13). Public health messages from public health authorities may also affect public perceptions of the crisis (14). Thus, the way in which the media characterizes public health messages may potentially play a pivotal role in whether the public develop positive or negative reactions to the situation that has evolved around the COVID-19 pandemic. Population mental health and wellbeing may also be influenced by the level of trust people have in their government and health authorities, which in turn may be influenced by media coverage (15).

Evidence from previous pandemics suggests that the frequency and content of reporting can significantly influence health related attitudes and behaviors of individuals (16, 17). The role of the media is also crucial in health education by making information more available to the general public (18). The role of the media in communicating public health messages on mental health during COVID-19 is common in high-income settings (19). However, there is limited evidence about the role of the media on mental health in low-income settings (20).

Therefore, the primary aim of this paper is to present an analysis of mental health issues reported in online news media and print newspapers in Bangladesh during the first year of the COVID-19 pandemic. This covers the period from 8 March 2020 when the first case of COVID-19 in Bangladesh was identified (10). Although we focus here on Bangladesh, we have noted the rapid escalation from case-identification to lockdown is not unique. Gaining a better understanding of the extent to which mental health was covered during this phase of the pandemic in Bangladesh, as well as the way in which it was covered, can help governments with future public mental health communication strategies, including their direct interactions with the media. This may be particularly important if poor mental health means that populations do not adhere to stringent public health guidance including social distancing and restricted travel that are necessary to contain any infectious disease outbreak. The secondary aim of the paper is to identify local initiatives undertaken by the government and nongovernmental organizations to support mental health during the pandemic as reported from the viewpoint of media.

## Methodology

### Sample Selection and Data Sources

We conducted a document review of seven Bangla and three English language daily newspapers (Table 1) between 8 March, 2020 (when the first COVID-19 case was reported) and 31 March, 2021. The reason for choosing these papers is because they have the highest circulation figures in Bangladesh (10, 21).

**Table 1:** List of searched newspapers and news portals.

**English**	**Type**	**Bangla**	**Type**
New age	Newspaper (online)	Prothom Alo	Newspaper (online)
The Daily Star	Newspaper (online)	Daily Kaler Kantha	Newspaper (online)
Dhaka Tribune	Online news portal	Daily Janakantha	Newspaper (online)
		Bangla Tribune	Online news portal
		Bdnews24.com	Online news portal
		Banglanews24.com	Online news portal
		Jagonews24.com	Online news portal

### News Coverage Identification

Through a series of iterative discussions, we deliberated on different factors that had been potentially associated with changes in mental health during COVID-19, proposed key themes to focus on, and discussed these further before agreeing on four news themes which we felt would be particularly relevant to examine (Table 2). We used the socio-determinant of mental health framework (22) as a starting point in discussions, looking to see which factors would be likely to impact on mental health in Bangladesh during pandemic conditions. These included impacts of mental stress, potentially linked to uncertainty over jobs and livelihoods, as well as access to informal and other support (23, 24). This uncertainty has been associated with poor mental health during the pandemic. A second theme we agreed on was suicide; at the start of the pandemic internationally concerns were raised about potential increased risks of suicide (25), and poor newspaper reporting has been linked to suicidal acts (26). Women have also been highlighted as potentially one of the most vulnerable populations during the pandemic (12); finding themselves having to both work and look after families and being at increased risk of domestic violence during periods of lockdown (27). Our final agreed theme was the extent to which positive news stories on mental health support were featured in the press; again we noted that stigma around mental health may act as a barrier to even looking for support in normal circumstances (28), and felt that during a pandemic it would potentially be even more important to increase awareness to help and support through the media.

**Table 2:** Operational definitions of the themes from reports.

**Themes**	**Definition**
***Theme 1: Mental stress due to COVID-19***	News reported any type of mental health stress amid COVID-19 situation for different population and occupation groups (such as garment workers, health workers, retired person etc.) in Bangladesh
***Theme 2: Suicidal behavior during COVID-19***	Any reported news of suicides between March, 2020 and March, 2021 amid the COVID-19 situation
***Theme 3: Impacts on working women and their families***	News reporting the impact of COVID-19 among working women in Bangladesh. Domestic violence including sexual violence reported within the reported period (March, 2020–March, 2021). Also, initiatives that have been taken by different government and nongovernment organizations to mitigate the burden of domestic violence
***Theme 4: Mental health support/ advice shared by other non-government organizations/ institutes or mental health experts***	News reporting on mental health supports that have been initiated by government, and non-government organizations through mental health experts

### Scoping the Dataset

**Figure 1:**
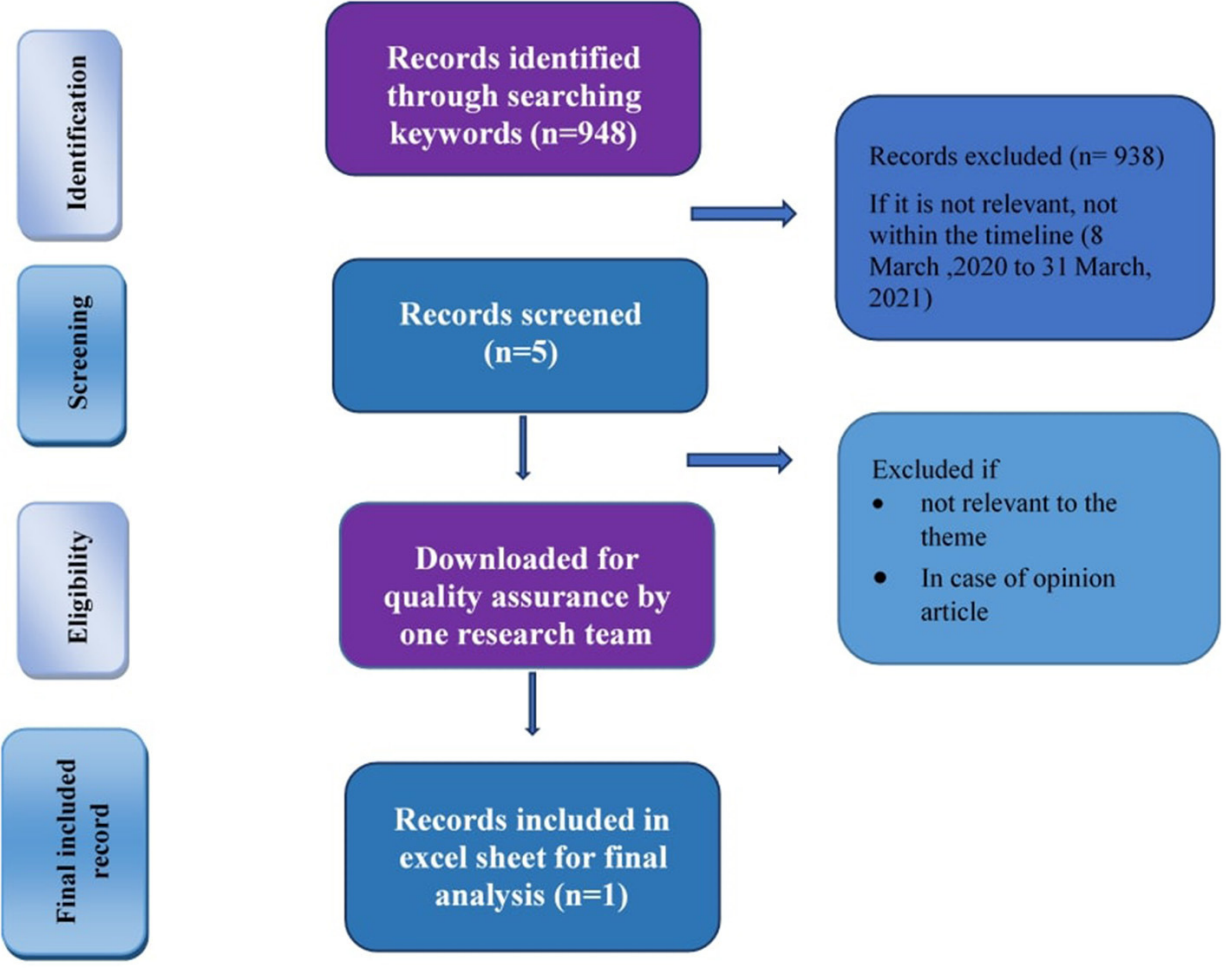
Flow chart showing strategy adapted for one search term (Mental stress and COVID-19) in one Bangla newspaper (Daily Kaler Kantho).

Four authors (MNA, NC, NF, and SI) conducted searches within the selected newspapers based on these predefined themes, with additional search terms and topics picked up during preliminary analyses of search results (Table 3). Firstly, each team member using search terms for predefined themes went through the search engine of each newspaper. For each search term, we received around 10 links per page and on average 15 pages per search term. Two authors (MNA and NC) screened the records and excluded news if it was not relevant as per the study objective and not within the timeline of review (8 March 2020 to 31 March 2021). For quality assurance, one author (NC) downloaded the selected news and excluded the opinion articles and reports that did not meet criteria of the themes. Selected news articles from all the selected newspapers were compiled in an Excel spreadsheet including the news title, access link, date of publication, search topic, searched by, search date, key messages and the translation of information in Bangla newspapers into English. These news stories were then categorized using the specific topic areas and sub themes. The process has been explained in Figure 1 for one search term under Theme 1: Mental stress due to COVID-19 from one newspaper; Daily Kaler Kantho. The same process has been executed for all themes and all selected newspapers (Figure 1).

**Table 3:** List of searched topics.

**Search terms and topics**	
**For English newspapers**	**For Bangla newspapers (translated)**
Mental stress and COVID-19	Mental stress is increasing during lockdown
Suicide during COVID-19	Domestic violence during COVID
Mental health of kids during lockdown	Suicide during COVID-19
Domestic violence during COVID-19	Mental stress and COVID-19
Harassment faced by corona patients	Funerals during COVID-19
Mental health services during COVID-19	Mental health services during COVID-19
Care of mental health during lockdown	

### Data Analysis and Quality Assurance

We undertook a mixed methods analysis where we documented the number of publications of mental health related stories and then assigned stories to themes/sub-themes (29). Quantitatively, we calculated descriptive statistics to identify the frequency of news reports under each theme in different newspapers. We have reported the proportion of primary outcomes in percentages (30). For qualitative work, we analyzed the data using a framework analysis (29). We prepared a matrix based on the pre-defined themes. Researchers read through all the newspapers published during the given time period, and inserted key messages and quotes into the matrix as per themes. The data was systematically categorized by identifying themes or patterns for smaller units or codes (31). This coding process employed both inductive and deductive coding, which included codes either predetermined or emerging from the data (32). A senior author (AN) who was not involved in the process of news searching, randomly checked all the news reports and identified any news item that was not aligned with the selected themes. All authors summarized the themes, highlighted the key messages and compared the contents. One author (MNA) counted the number of reports under each theme to estimate the coverage that each theme retrieved. Through iterative deliberative discussion the authors agreed on highlighted quotes from different media sources to support these themes.

## Results

### Pattern of News Coverage on Mental Health Issues in COVID-19

A total of 201 news items (reports) were identified during the study timeframe. The highest number of news items were reported in April of 2020 at the time of the peak of the first year of COVID-19 and a higher number of news items was reported in March 2021 in the second year of COVID-19 pandemic in Bangladesh (33). We have collated these news reports in an Excel spreadsheet and sorted them out in to four news coverage themes based on the reports on different patterns of mental health issues during COVID-19, including mental stress and suicide. Each of the four news coverage themes identified are now discussed.

#### Theme 1: Mental Stress Due to COVID-19

We gathered 45 articles (22.4%) out of 201 reports from nine newspapers that focused on mental stress and COVID-19. This included reports from six studies conducted early in the pandemic in Bangladesh that explored its impact on the general mental health and well-being of the population. Among them four were peer reviewed and two were published through an online institution portal (34–40). The newspapers also reported that the health workers, garment workers and the urban poor, living in informal settlements, were struggling to cope up with the sudden impact of the COVID-19 (34).

One report covered a multiphase phone survey conducted in April 2020 with 1,309 participants that found that stress was associated with loss of income. Fifty-eight percent (58%) of people who had lost all of their income reported being stressed compared to 29% of people who had lost only a portion of their income, and 13% of people who had no income loss. This was coherent with the reports published on the institutional website of the research study team (5, 34, 40). The Daily Star, the most circulated English newspaper, reported on a study that highlighted that the majority of anxiety experienced by families was mainly due to uncertainty caused by COVID-19. The same newspaper reported that in the capital city, Dhaka, about 80 case studies were being conducted to explore daily experiences, as well as the emotional and mental well-being, of inhabitants of six slums located in some selected city corporation areas (local governing administrative bodies). Respondents reported that they experienced panic, restlessness, hopelessness and fear, as well as occasional break downs due to grief (41).

An online English news portal bdnews24.com also reported on the situation faced by older people. The report reflected on their loneliness, given they could not go outside or have their relatives visit them during the lockdown (42);

“*Before the lockdown, my grandchildren used to meet me almost every day. At this stage of my life they are my source of happiness. But now I cannot get a chance to meet them***”** (bdnews24.com translated into English, 25 April, 2020).

Another news item in the same newspaper reported that some older people who lived with their families expressed concern about the health of working family members, although they themselves were not worried about loneliness. One older man reported that he was more scared of the virus than the Bangladesh Liberation War, describing it as an invisible enemy;

“*I don't feel safe at home. It is uncertain when the invisible virus will attack us as my family members are going outside. I have seen the liberation war of 1971. That was with a known enemy. But this war is even worse, as the enemy is invisible and there is no antidote yet*” (bdnews24.com translated into English, 6 April 2020).

Eight news reports from six newspapers (New Age, Banglanews24.com, Daily Janakantha, Daily Prothom Alo, Jagonews24.com, and Bangla Tribune), reflected on the risk of people falling into poverty because of the COVID-19. Day laborer's reported being stressed due to income insecurity; many had already become jobless and were unsure about the future. Garment workers were also stressed and worried about losing their jobs. The President of the Bangladesh Economic Association, was quoted in one of the newspapers,

“*I would say, those who suffer the most due to COVID-19 are the poor. If the infection rate increases, then the middle class will also be in trouble. Those who are day laborer's, if they cannot work, they will starve; and if the price of goods goes up there is nothing they can do. Added to that, having to wash your hands with soap creates an extra [monetary] burden*” (Daily Janakantha translated into English, 23 March, 2020).

Doctors and other frontline health workers were also reported to be stressed due to the lack of personal protective equipment (PPE). An English newspaper, Dhaka Tribune, reported that health workers were experiencing immense stress, thinking that their families would be at risk of infection because of their potential exposure to COVID-19 patients at work (43). A report from Jagonews24.com (18 April, 2020) focused on the stress of pregnant nurses who were still providing services (44). A husband quoted; “*because my wife (nurse) is pregnant, she informed her supervisor that she needed to stop working, but her supervisor said that given the current situation this was not possible.” He [the husband] added that his wife was mentally broken and worried about the severe repercussions of COVID-19 on her unborn child*” (Jagonews24.com translated into English, 18th April, 2020). Another report from the Daily Star revealed that around 18% of physicians and 7% of the allied health professional had depression (45).

More recently the Prothom Alo referred to a study report conducted among 2,037 laboratory confirmed COVID-19 patients between the ages of 20 and 60 years highlighting that around 85% of people suffer from severe anxiety, 17% suffer from severe depression and 53% of patient have a tendency of partial memory loss (46). The Dhaka Tribune also reported a study that showed around 81% of the transgender (*hijra*^1^) community and other gender-diverse populations of Bangladesh were struggling financially and facing food crisis. The article reported one survey, conducted among 88 members of the transgender community that found 93% experienced mental stress and anxiety, as well as food and financial insecurity, related to the lockdown. These pressures compounded existing forms of stigma and discrimination. The same article also reported the positive news that the Bandhu Social Welfare Society (BSWS), which supports the transgender and gender-diverse population of Bangladesh, provided contact numbers for mental health counseling and were working to improve emergency medical services for these populations (47).

#### Theme 2: Suicidal Behavior During COVID-19

One of the major issues reflected in the newspapers was suicide which is a public health issue globally, particularly in Asia where there are high suicide rates in several populous countries (10). There is no official surveillance system for suicide in Bangladesh and newspapers have long relied on police, court, and other media documents when preparing stories on apparent suicides (48).

**Figure 2:**
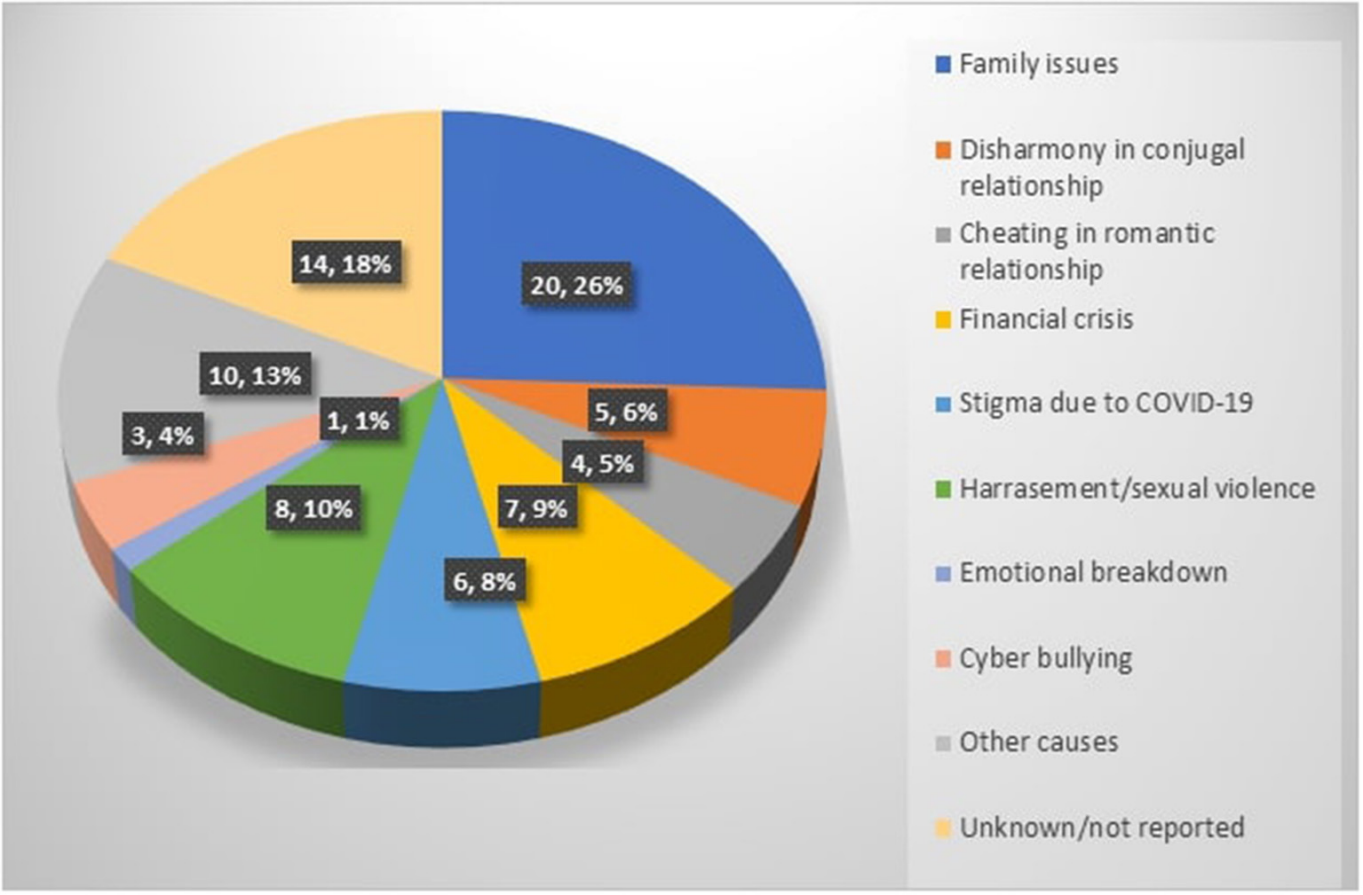
Factors linked to newspaper reported suicidal events during COVID-19.

In total we have identified 50 (24.9%) articles out of 201 news articles published in 7 newspapers (Daily Janakantha, Bangla Tribune, Jagonews24.com, Prothom Alo, Daily Kaler Kantho, Dhaka Tribune, and The Daily Star) (Figure 2), which referred to 80 reports of potential suicides (32 male, 48 female). Among them 43 people were 10–24 years old, 20 people were 25–35 years old and 10 were above 35 years old, including 4 people above 60 years old. Most of these suicide cases (*n* = 20) were reported to be due to family issues and they were mostly reported in the Jessore district in South-western Bangladesh (n=12). We have highlighted the possible causes of suicides reported by media (Figure 2). Among this one report narrated the suicide of a young housewife who had an argument with her expatriate husband over a video call regarding the donation of financial aid to help people affected by the pandemic. The papers reported that she died as a result of suicide whilst on the video call with her husband (49).

Seven suicide cases were reported to be related to a financial crisis; these included the case of a young couple who could not pay debts as quoted by the Additional Superintendent of Police below;

“*We have spoken with local people and relatives. They reported that they took loans from different NGOs to build their house. However, due to the COVID-19 situation they might feel the burden of debt and committed suicide*” (Bangla Tribune translated into English, 7 June, 2020).

In total six possible suicides (5 male, 1 female) were reported by the newspapers as being linked to COVID-19. One person was reported to have escaped from hospital after being diagnosed with COVID-19 before dying through suicide (50). “*He called his wife that night and said that he was about to die in a little while. He was in great pain. When his wife asked what happened he hung up the phone. We went to the hospital the next day to find him and came to know that he escaped from there and was diagnosed with Corona and that day he committed suicide*” (Shomoy news, translated into English 21 June, 2020). Two possible suicides were due to negative attitudes of people towards COVID-19 patients. One case was of a police officer who was subjected to social stigma even after he had tested negative for COVID-19 (51). The duty police officer was quoted as saying;

“*He tested negative but yet his family members and neighbors suspected that he was* COVID-19 *positive and started avoiding him. He broke down mentally, didn't even eat properly for the last few days*” (Collected from Dhaka Tribune, 4 May, 2020).

Six suicide cases among married women were reported to be due to disharmony in their conjugal relationships. These might be because of the stress regarding uncertainty of future, a lack of personal space and the inability to do normal activities due to lockdown during the pandemic situation. The media also reported eight suicides among young women due to harassment and sexual violence. Of them, one case concerned a young housewife who was raped by her brother-in-law and later died by suicide. The article also reported the method of suicide, something that is not recommended internationally (52). Three women were reported to have died from suicide as a result of sexual harassment arising from the viral circulation *via* social media of sexual images, obscene conversations and other offensive pictures.

We also documented eighteen suicide cases among adolescents and young adults (12 female and 6 male) due to emotional breakdown amid this COVID-19 situation. Six were reported due to being unsuccessful in secondary school board exams (final examination), five to being upset with their families, three being cheated in romantic relationships, three were due to grief of not obtaining things that they wanted and one due to severe anxiety triggered by her father contracting COVID-19 while working in Qatar. Her grandmother quoted;

“*Her father was under treatment for COVID-19 in Qatar, besides he also had kidney failure. He used to talk to us every 4 days. Her father loved her so much and being worried about him, she committed suicide out of frustration*.” (Jagonews24.com translated into English, 29 May, 2020).

In addition, we have identified 13 apparent cases of suicide where the actual causes were not reported in the media. There were also two reported cases of suicide among older people due to loneliness and grief at being assaulted due to political issues.

#### Theme 3: Impacts on Working Women and Their Families

We gathered 77 articles (38.3%) from nine newspapers (New Age, Bdnews24.com, Bangla Tribune, The Daily Star, Dhaka Tribune, Prothom Alo, Daily Kaler Kantho, Jagonews24.com, and banglanews24.com) that focused on impacts on working women and their families.

##### Burden of Traditional Roles in Family During COVID-19

The current COVID-19 situation has exacerbated the burden of household chores especially for working women who had to manage both paid work and household chores simultaneously.

News media have also highlighted the double burden on working women during the COVID-19 pandemic due to gender inequalities (2 out of 77 reports, 2.5%). Working mothers in particular, experienced over burden from having to juggle paid work with all household chores, in a situation where paid housekeepers were no longer permitted on public health grounds. Bdnews24.com in April 2020 also reported that working women in Bangladesh were having to deal with stress in the office, as well as at home, without recourse to help from paid housekeepers or support from other family members (53). One woman was quoted as saying;

“*I [had] housekeeper before the lockdown. But now, I have to work from home and she [the housekeeper] is not even allowed to come to my home. I have to do office work on time, on the other hand I also have housework*” (bdnews24.com, 3rd April, 2020).

##### Domestic Violence During COVID-19

An increase in domestic violence directed towards women and children during the pandemic is another issue that has been highlighted in the newspapers (63 out of 77 news reports, 82%). This includes one report in the Bangla Tribune on 10 June 2020 regarding a study that showed approximately 11,025 women suffered domestic violence during the countrywide shutdown, including 4,947 women subjected to psychological abuse and 3,589 abused due to financial matters (54). The Bangla Tribune on 6th May, 2020 reported comments from the Executive Director of Manusher Jonno Foundation (MJF) who stated that women who had never been accustomed to any previous domestic violence were reporting domestic violence during this lockdown. The Dhaka Tribune also reported that around 40,000 women had faced domestic violence between April and September 2020, with 40% experiencing this for the first time (55). In another report the Dhaka Tribune referred to a study highlighting that about half of respondents blamed their spouses for being responsible for the increased violence inside the family (56). The Daily Star referred to one study conducted in 240 unions (small administrative areas) of 54 sub-districts within 18 districts of the country which reported that around 4,550 families, including children and women, were experiencing domestic violence (57).

##### Sexual Violence Against Women and Children

Sexual violence towards women and/or children was reported in four newspapers (Daily Janakantha, Daily Kaler Kantho, Bangla Tribune, and The Daily Star) covered by 12 news reports out of 77 reports (15.5%). The Daily Kaler Kantho reported a statement from the Bangladesh Women's Council who said that around 3,440 women and children had been raped and tortured during the COVID-19 period in 2020 (58). The Bangla Tribune covered another study that reported on 179 victims of sexual harassment, as well as 48 cases of rape or attempted rape during financial relief collection (54).

#### Theme 4: Mental Health Support/Advice Shared by Other Non-government Organizations/Institutes or Mental Health Experts

Twenty-nine (14.4%) news articles in seven newspapers (Bangla Tribune, Dhaka Tribune, Banglanews24.com Bdnews24.com, Jagonews24, The Daily Star, and Daily Kaler Kantho) reported on several government and non-governmental organizations that have taken initiatives to provide mental health support for vulnerable populations such as the older age population, children, and women. The initiatives included “Kaan Pete Roi,” an emotional support telephone service that planned to provide emergency tele-counseling support for people experiencing distress, frustration, and stress caused by the COVID-19 pandemic (59). However, a newspaper shared results of a recent online survey of Kaan Pete Roi which reported a 60% decrease in client calls due to their home circumstances, for instance not being able to discuss their issues without being overheard, and also due to difficulties bearing the costs of mobile phone calls (60, 61).

Other initiatives reported in the media also included expert counseling from platforms such as “Moner Jotno Mobile E” that provide essential mental health support to individuals, so that they can better tackle this crisis and inspire a sense of hopefulness about the future (62). Another example reported on tele-counseling for the elderly, chronically ill, and children with special needs (63).

We highlighted earlier media coverage of the impact of the pandemic on garment workers. The media have also reported that the Bangladesh Garment Manufacturers and Exporters Association (BGMEA) and Maya (an online service providing access to professional mental health support) signed an agreement to provide free services to help prevent and limit the spread of COVID-19, while also addressing general medical and mental health related issues amongst ready-made garment (RMG) workers (64, 65). Additionally, an online platform “Moner Daktar” comprising a website and a mobile app, has been developed where 72 psychologists voluntarily provide psychological assistance and mental support services (66, 67).

The Bangla Tribune also reported on an initiative to mitigate domestic violence by the Socialist Women's Forum. They delivered a memorandum to the Home Minister's office at the Secretariat (Government Buildings) demanding an end to violence against women and ensuring women's safety during the COVID-19 situation (68).

## Discussion

This review looks at the impact of COVID-19 on mental health, as well as initiatives to protect mental health, through the lens of mainstream newspaper media coverage in Bangladesh. Our analysis is limited solely to the newspapers and online news portals rather than traditional radio or television broadcasts, as well as social media. These other forms of media may provide different content and perspectives. However, we have analyzed the content of the top 10 newspaper circulation media covering both Bangla and English publications and these have a combined daily circulation of around 205,556. Our analysis is also subject to reporting bias; news media typically puts more focus on adverse rather than positive events. News reports also tend to be short in length and often do not provide detailed information on the studies that they choose to highlight. This limits our content analysis.

Our findings highlight important insights from newspapers on perceived key mental health issues during COVID-19 that includes mental stress due to loss of income, something that has been reported in other LMICs such as India (69). They are also consistent with findings of a population survey that revealed the adverse impact of COVID-19 on the mental health of general population in Bangladesh (70). Loneliness was another important factor in media coverage that has been mostly reported by older people, one of the most vulnerable populations in the country. There is also emerging evidence on this issue in other countries; according to a general population survey conducted in UK, long term isolation or quarantine has severe repercussion on mental health wellbeing (71).

The psychological health of doctors and nurses on the health care frontline has been another focus for the media. They report medical staff to be suffering from stress and anxiety due to the COVID 19 situation. This situation is also seen in many countries. For instance, a team of researchers from the USA reported that health care providers were stressed by the lack of personal protective equipment (PPE) and the fear that while serving the country their family members will also be infected by Coronavirus (72). In Bangladesh media coverage has included reports on pregnant health care providers anxious about the impact of COVID-19 on their own unborn children.

The media have a critical role to play in communicating accurately on COVID-19 and the necessary health system responses. The “infodemic” or “overabundance of information and the rapid spread of misleading or fabricated news, images, and videos” that the WHO has highlighted throughout 2020 can be fueled further if misleading stories are covered by print and online newspapers (73). Global analysis of online media at the very start of the pandemic until April 5 has reported a high level of misinformation across many countries, including some rumor and stigma in Bangladesh (5). Fake or misleading news can be shared instantly in the social media causing confusion and panic in the community (74).

However, Bangladeshi newspapers can also reflect some positive news and our review also reflected on how the media has presented the findings of different studies conducted during COVID-19 to make people aware about mental stress. This includes risks from domestic violence. However, there has been no general principle on assessing robustness of the method of any studies. While some of stories draw on university-based research, most of these reports rely on unpublished data and news articles; typically do not contain adequate information to check the veracity of credible information to support the findings. One positive aspect of newspaper reporting of mental health issues is that these are reported along with the details of available services for supporting mental health care; however, it was not common in case of suicidal news reporting by newspapers in general.

Before the pandemic a study conducted by Action Aid Bangladesh showed that Bangladeshi women on average spend 6 hour a day on unpaid household chores including cooking, cleaning, and taking care of children and older people, while men spend no more than an hour on these activities (75). According to the United Nations Entity for Gender Equality and the Empowerment of Women (UN Women), domestic violence particularly towards women and children has increased during the lockdown (76). For instance in France, the lockdown was followed by a 30% rise in domestic violence (77). Our newspaper review found media coverage for studies conducted in Bangladesh that revealed psychosocial abuse and violence due to financial issues. Surprisingly, those who never previously reported domestic violence were willing to speak of increased risk of violence during this lockdown in two newspapers. This has been also reported in a study conducted in Bangladesh (78).

The media can potentially play a very important role, not only in clearly communicating and reinforcing public health messages, but also in highlighting potential sources of government and wider support to help people during the pandemic. This study has generated important evidence on the mental health issues that have arisen during the lockdown and initiatives that have been taken to fight the epidemic situation. The media did report on some measures to mitigate these mental health issues during COVID-19 situation. They highlighted actions by the government, as well as the non-governmental organizations, to provide tele-counseling and online mental health support. However, one of the evaluation surveys conducted by Kaan Pete Roi reported by the media noted that during the lockdown, that as all family members have to stay at home, clients cannot talk freely and have reduced use of services. Moreover, the newspapers have highlighted the high cost of mobile phone calls and data usage as another remarkable challenge to availing online support.

Another notable findings of our review was that 24.8% (50 of all 201 news articles) were focused on suicide, particularly among the adolescents and youth. In Bangladesh suicide cases are mostly reported in the newspapers based on local police reports. A content analysis of the media reporting suicide death in Bangladesh revealed that the mean number of suicide articles per day per newspaper was 0.3 with the mean length of 11.3 sentences (79). One of the important issues captured within this study was that around 75.5% had representation of harmful reporting practices whereas no articles are found to share details of suicide support services (79).

This newspaper reporting of suicide is potentially concerning. Newspapers provide individual examples of potential deaths through suicide during this time period, but this does not provide objective information on whether suicide rates are increasing or decreasing. This can only be done when detailed statistical information on completed suicides become available and can be compared, for instance, with average suicide rates at the same time of year in previous years. We can though point to some examples from previous outbreaks such as during Acute Respiratory Syndrome (SARS) in Hong Kong, when Chan et al. reported a significant increase of suicidal death in some vulnerable population groups (80).

How suspected suicides are reported is another issue of concern that every newspaper needs to be careful of. A content analysis conducted in Bangladesh and India revealed that there is an imbalance in reporting the suicide cases that often detailed information on the process and means of suicide (81). This potentially could result in severe repercussions in the population, including among people living with poor mental health. There is evidence internationally of an association between suicide reporting and increased future risk of suicides in the population, especially when suicide involves high profile individuals such as sports people, politicians or entertainers (26, 82). Newspapers can though help to reduce the risks of suicidal behavior by providing information on helplines and other supports for people in distress (70). However, newspaper reports in Bangladesh do not aim to educate people about suicide, including information on where to seek care during any mental health emergency. Risks of self-harm and suicide may also be fueled further if misleading stories are covered by print and online newspapers. One major newspaper reported the findings of a study that collated materials from three daily newspapers, 19 local newspapers, hospitals, and police records suggesting that 14,436 persons died as a result of suicide between March 2020 to February 2021; however, there was no detailed information or reference of the study procedure in the media report to verify the study report (83).

We have noted that the media's portrayal of the mental health consequences of the pandemic could itself have an adverse or positive impact on mental health. The media has the potential to break the stigma and raise awareness during any pandemic situation (84). Media such as newspapers can act as a bridge between government and society especially during a crisis like COVID-19. In this critical situation, the media could play an important role sensitizing and contextualizing the situation to the public for motivating them to engage in positive actions (85). Responsible reporting by the media about the role of the society towards mental health in COVID-19 can help policymakers plan strategies for mental health care for adopting timely public health measures and promoting local organizations averting the risk of negative consequences relevant to mental health issues while supporting physical, mental, and social wellbeing of the people, particularly the youth during the pandemic (86, 87).

## Conclusion

Our analysis of newspaper media reports indicates that COVID-19 has had severe repercussions for the mental health of the general population, as well as high risk groups including children, older people and garment industry workers. Reports suggest an increase in mental health problems such as stress, as well as suicide and domestic violence.

Positively, the news media have also highlighted the mental health supports initiated by the government and non-governmental organizations to promote better mental health. This can help in raising awareness and promoting the use of these services. News media can also have influence on policymaker's perceptions of the COVID-19 situation. Changes in public mood may be reflected and news stories help to identify how public behavior is changing. In low and middle-income country settings, where real time surveillance data may be limited, if carefully contextualized this information can be used to help policy makers further plan responses to the pandemic, including the roll out of vaccinations.

There are also insights on the potential role of the media in reporting any pandemic. Government could work more in partnership with media to communicate and reinforce public health messages and also raise awareness of risks to mental health, as well as mental health supports that are available. Measures might also be taken to improve capacity in the news media on the way in which they report mental health issues, including suicide, during major health crises. Better communication of the potential risks of infection, and ways in which any virus is transmitted, can also help tackle some of the anxiety and stigma associated with a new infectious disease.

## Data Availability Statement

The original contributions presented in the study are included in the article, figure, and supplementary table, further inquiries can be directed to the corresponding author/s.

## Author Contributions

AN conceived the study. AN and DM contributed in conceptualization. NC, NF, and SI supported in data curation with supervision of AN and TM helped in extrapolation of specific data. HJ contributed to prepare the data analysis section. MN cleaned and managed the data and drafted the first version of the manuscript. AN contributed in quality assurance and substantially refined the manuscript. AN, DM, and HJ guided her to prepare the manuscript. MU, HU, HJ, and DM critically reviewed the manuscript. All authors checked the facts thoroughly and approved the final version of the manuscript.

## Author Disclaimer

The views expressed in this publication are those of the author(s) and not necessarily those of the NHS, the National Institute for Health Research or the Department of Health and Social Care.

## Conflict of Interest

The authors declare that the research was conducted in the absence of any commercial or financial relationships that could be construed as a potential conflict of interest.

## Publisher's Note

All claims expressed in this article are solely those of the authors and do not necessarily represent those of their affiliated organizations, or those of the publisher, the editors and the reviewers. Any product that may be evaluated in this article, or claim that may be made by its manufacturer, is not guaranteed or endorsed by the publisher.

## Footnotes

^1^The term “hijra” is the term used in Bangladesh (and other South Asian countries) for someone born male but having a feminine gender identity. They are sometimes referred to as “third gender”.
